# Improving Blind Docking in DOCK6 through an Automated Preliminary Fragment Probing Strategy

**DOI:** 10.3390/molecules26051224

**Published:** 2021-02-25

**Authors:** Paula Jofily, Pedro G. Pascutti, Pedro H. M. Torres

**Affiliations:** Laboratório de Modelagem e Dinâmica Molecular, Instituto de Biofísica Carlos Chagas Filho, Universidade Federal do Rio de Janeiro, Rio de Janeiro, RJ 21941-902, Brazil; paula.jofily@biof.ufrj.br (P.J.); pascutti@biof.ufrj.br (P.G.P.)

**Keywords:** blind docking, ftmap, dock6, pipeline

## Abstract

Probing protein surfaces to accurately predict the binding site and conformation of a small molecule is a challenge currently addressed through mainly two different approaches: blind docking and cavity detection-guided docking. Although cavity detection-guided blind docking has yielded high success rates, it is less practical when a large number of molecules must be screened against many detected binding sites. On the other hand, blind docking allows for simultaneous search of the whole protein surface, which however entails the loss of accuracy and speed. To bridge this gap, in this study, we developed and tested BLinDPyPr, an automated pipeline which uses FTMap and DOCK6 to perform a hybrid blind docking strategy. Through our algorithm, FTMap docked probe clusters are converted into DOCK6 spheres for determining binding regions. Because these spheres are solely derived from FTMap probes, their locations are contained in and specific to multiple potential binding pockets, which become the regions that are simultaneously probed and chosen by the search algorithm based on the properties of each candidate ligand. This method yields pose prediction results (45.2–54.3% success rates) comparable to those of site-specific docking with the classic DOCK6 workflow (49.7–54.3%) and is half as time-consuming as the conventional blind docking method with DOCK6.

## 1. Introduction

Computer-aided drug design (CADD) comprises in silico methods for simulation, visualization, and prediction of biological phenomena. Such methods facilitate, accelerate, and even enable drug discovery. To this end, it is of great importance to accurately predict possible interactions between protein targets and small organic molecules. In this field, molecular docking and virtual screening are valuable tools which allow for fast binding pose prediction and scoring of the protein–ligand complexes [1].

It is generally accepted that binding affinity prediction and scoring present greater challenges than pose generation (posing) [2], thus pose prediction remains critical for interaction studies and directly impacts scoring and ranking. Furthermore, when the ideal protein binding site is not known or specified, pose prediction becomes a greater challenge, since in these cases computer methods must search the entire protein surface for the correct binding pose through the strategy known as blind docking.

In light of this, two main distinct strategies to perform blind docking have arisen: (*i*) In conventional blind docking, the docking box is extended to encompass the whole protein, and the docking algorithm itself must probe the whole receptor surface when performing pose prediction [3,4,5]. (*ii*) In cavity detection-guided blind docking, a separate algorithm identifies and ranks possible binding pockets a priori. Docking is then performed at the site(s) identified as most probable [6,7].

Most commonly, blind docking has been performed using established docking programs such as AutoDock [8], AutoDock Vina [9], GOLD [10], and Glide [11] and molecular docking servers such as DockThor [12] whose algorithms were not specifically developed to tackle blind docking challenges. Nevertheless, these tools have been employed in blind docking with relative success in situations where the docking box encompasses the whole receptor surface [4,5]. Furthermore, to aid in cavity detection-guided blind docking, many methods for cavity detection and characterization have been developed to this date, such as FTSite [13], CavityPlus [14], DoGSiteScorer [15], DeepSite [16], FPocket [17], and PocketZebra [18]. Recently, tools and servers which are specifically tailored to perform and automate blind docking, such as SwissDock [19], COACH-D [6], PatchDock (for rigid docking) [20], BSP-SLIM [21], QuickVina-W [3], and CB-Dock [7], have also been developed.

Conventional blind docking is most challenging accuracy-wise, because of the increased amount of binding possibilities the docking algorithm must account for and evaluate. However, especially in a virtual screening circumstance, this method carries a big advantage: it may be able to determine, for each candidate ligand, the best binding pocket in a single docking run. Cavity-detection guided blind docking greatly increases pose prediction performance, since cavity selection reduces the search space required of the docking algorithm. On the other hand, if more than one of the detected sites is required for interaction studies, one docking or virtual screening run must be performed for each of the desired sites [6,7,22]. This can be computationally expensive and time consuming, especially in situations where the candidate ligands are numerous. Furthermore, in these cases, more intense post-processing is necessary to merge the results and thus final analysis may be less comprehensive [3,7].

Interestingly, the SwissDock web server [19], with EADock DSS [23], implements a method which unites traits from both approaches by employing a cavity detection algorithm which singles out the most promising cavity points for pose prediction. Its context as a web server, however, makes it not amenable for screening larger ligand libraries.

Hence, there is a gap of accuracy versus speed and practicality between approaches, especially for screening larger libraries, which often requires a compromise in accuracy which hinders ranking enrichment. To aid in bridging this gap, we seek to unite the speed and practicality of blind docking with the advantages of cavity detection in a single automated pipeline. In this study, we develop a hybrid approach in which multiple potential cavities are detected and specified in a cavity-detection guided manner, thus reducing the search space; however, such cavities can be sampled simultaneously for each candidate ligand, in a conventional blind docking manner. The pipeline, called BLinDPyPr (blind ligand docking through preliminary probing), is freely available for download at https://github.com/PaulaJLR/BLinDPyPr (accessed on 11 December 2020).

## 2. Methods

### 2.1. Pipeline Components

BLinDPyPr consists of a python program which automates a pipeline to access well established computational tools; and to combine them with conversion scripts in order to perform hybrid blind docking. The FTMap server [24] is used to probe the target protein’s surface and determine the most probable binding pockets. Schrödinger PyMOL is employed to perform conversions between molecular formats (such as .pdb to .mol2) and UCSF Chimera [25] is operated by BLinDPyPr to prepare receptor files for docking. UCSF DOCK6 [26] (versions 6.8 or later are compatible) is employed for obtaining pharmacophore definitions and for the molecular docking process.

### 2.2. Pipeline Workflow

#### 2.2.1. File Preparation

Users must provide BLinDPyPr with a ligand .mol2 file (or a multi .mol2, for virtual screening) ready for docking. As for the receptor, BLinDPyPr can receive a protein .pdb file and submit it to FTMap through scripted online access. All the result files generated by FTMap are downloaded to the working directory and are automatically forwarded to the docking phase. On the other hand, it is also possible to previously run FTMap independently and provide BLinDPyPr with the resulting .pdb file, which contains the target protein as well as the docked probe crossclusters. This option is required for site-specific docking calculations, where it is necessary to visually select crossclusters in pockets of interest on the receptor surface (cf. Section 2.2.2).

The docking-ready receptor .mol2 file will be automatically generated from the FTMap pdb result: the protein is separated from the probes and is submitted to Chimera DockPrep tool. In the case the user prefers to use a tailored receptor file, containing, for instance, specific charges or nonstandard residues, BLinDPyPr can be configured to use such .mol2 file for docking. However, it is important to note that such characteristics will not be taken into consideration by FTMap, since its protocol automatically adds hydrogens and charges and removes nonstandard atoms. Therefore, the probes will be docked in these conditions, even if the ligand is docked in the custom, user-defined receptor.

#### 2.2.2. Cavity Definition

DOCK6 employs a sphere-based method to define the docking space. Spheres are described in an .sph file and can be placed anywhere in the receptor surface. They can be created automatically by DOCK6 tool *sphgen*, selected in a specific radius around a reference point by the tool *sphere_selector*, or added by hand in the .sph file. In a DOCK6 run, the docking box is created around all the spheres present in a receptor surface; however, ligands are only docked where there are spheres, even if the docking box encompasses the entire protein.

Using the concept of spheres, BLinDPyPr provides different options to specify the regions and pockets for docking. The spheres can be classified into two groups: the FTMap sphere group, which is the new method introduced by BLinDPyPr, and the classic sphere group, which is created using *sphgen* and is automated with BLinDPyPr.

**FTMap Derived Spheres**

**Figure 1:**
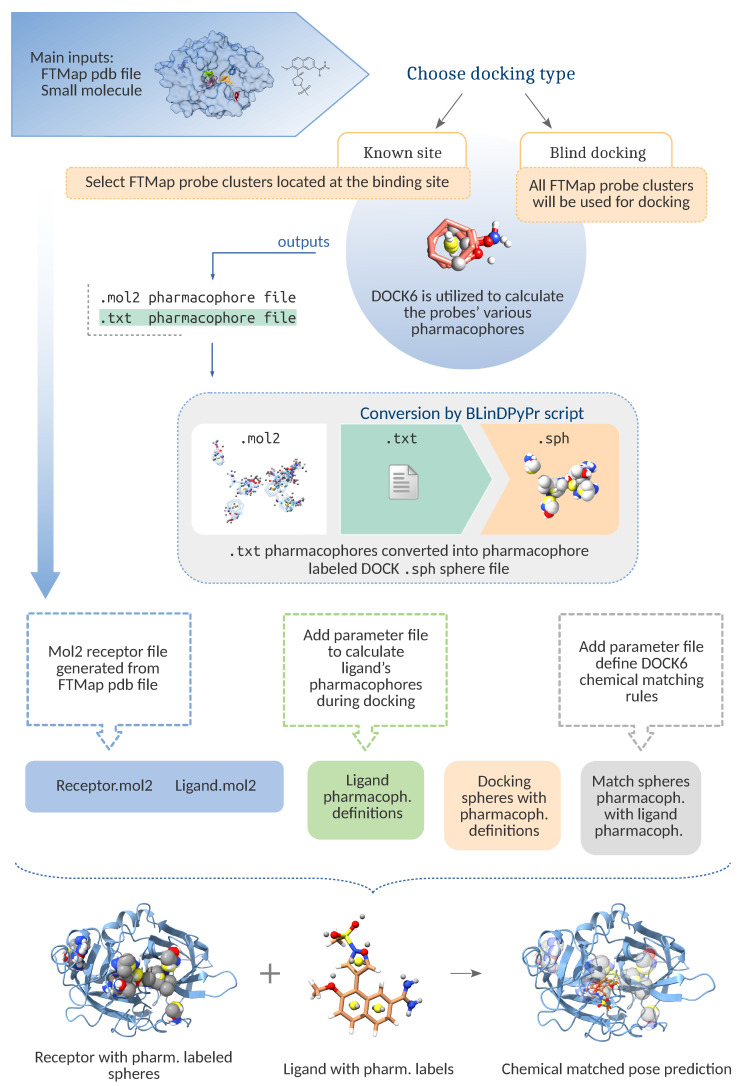
Schematic for the main conversion routine, which allows for multiple cavity guided blind docking with DOCK6. The figure also illustrates the chemical matching process which may be used to further orient the ligand. The docked probe crossclusters from FTMap PDB file are converted into mol2 format and fed to DOCK6 scoring function FMS to calculate pharmacophores. These are converted into the docking spheres necessary for a DOCK6 run, whose labels match the pharmacophores from which they were generated. If the user chooses not to perform chemical matching, BLinDPyPr will create a DOCK6 input with the *chemical_matching* flag turned off.

Here, FTMap probe crossclusters (the probe groups output by FTMap after final clustering) are converted into spheres, referred to in this manuscript as FTspheres. This means that the spheres will be restricted to the regions FTMap chooses as potential binding pockets. This increases the specificity of the search for a docking pose, therefore increasing the chance of an accurate pose prediction. The conversion is carried out as follows (Figure 1).

The BLinDPyPr script separates the probes from the main FTMap .pdb file and converts them into .mol2 format using PyMOL. These are then provided as input to the DOCK6 scoring function Pharmacophore Similarity Score (FMS), which is used to calculate the various pharmacophore definitions of the probes and to output them into a text file. The BLinDPyPr main conversion routine then converts the pharmacophore text file into a DOCK6 sphere file, along with information translated from the pharmacophore definitions themselves. If desired, chemical matching may be used to process this information and further orient the ligand: BLinDPyPr will replicate the pharmacophore parameter file utilized by FMS and provide it as input to DOCK6 so that it may find, in the candidate ligands, the same pharmacophore patterns it found for the FTMap probes. Additionally, it will create a correlation table instructing DOCK6 to match ligand pharmacophores to the ones found in the docking spheres’ labels. This matching occurs by discarding ligand conformations which produce unfavourable matches (i.e., a ligand pharmacophore overlaps with a sphere labeled for a different pharmacophore) [27].

Furthermore, users may choose any combination of FTMap crossclusters they wish by passing their numbers to BLinDPyPr. Only the desired crossclusters will be converted into spheres, guiding DOCK6 towards site-specific docking, multiple-site docking, or, if none are specified, BLinDPyPr will select all of them in order to perform blind docking.

This type of approach cannot be categorized in either of the previously mentioned blind docking approaches. The type of blind docking prepared by BLinDPyPr with FTspheres guides DOCK6 towards multiple potential binding sites robustly identified by FTMap, which prevents it from probing the whole protein surface, while at the same time allowing it to evaluate each candidate ligand in all the FTMap defined pockets, simultaneously, in a single virtual screening or docking run.

**Classic Spheres**

BLinDPyPr can also generate default spheres using the DOCK6 sphere generator (*sphgen*), which calculates spheres throughout the whole receptor surface and automatically clusters them, thus sphere Cluster 1 is the most probable to overlap with the real receptor binding site, while Cluster 0 is equivalent to the whole sphere set.

If the *sphgen* flag is passed to BLinDPyPr, it will refrain from generating spheres from the FTmap probes and will instead use the classic spheres. Users can select any *sphgen* sphere cluster. If Cluster 0 is selected, all spheres will be used for docking, which will consequently happen in a conventional blind docking manner, as previously discussed.

It is also possible to automatically select spheres using DOCK6 *sphere_selector* through BLinDPyPr. If the user analyses the FTMap results and identifies crossclusters of interest but needs classic spheres in that region, the numbers of the selected crossclusters may be passed in addition to the *sphgen* flag. In this case, BLinDPyPr will select spheres within a three Angstrom radius around them.

Independently of the sphere type created, BLinDPyPr runs DOCK6 program showsphere, which creates a .pdb from the sphere .sph file, so that it can be observed by the user through a visualization program.

#### 2.2.3. Docking Preparation

DOCK6 tool *sphgen* requires a receptor surface file, which BLinDPyPr will generate automatically through UCSF Chimera if the user opts for DOCK6 classic method. It is noteworthy that the surface component calculation with UCSF Chimera is challenging for some proteins. If there is an error in these calculations, impacting the integrity of the surface file, *sphgen* will not run successfully. Such errors do not occur with FTMap spheres since they are converted from the docked probe pharmacophores and their creation does not require surface file generation.

Following sphere generation, BLinDPyPr will define the docking box using the DOCK6 tool showbox, with a default margin of five Angstroms around the generated spheres. If desired, users can alter this value to any value of interest.

The grid calculations for the electrostatic and Van der Waals potentials in the docking box are automatically run through DOCK6 grid, with an input file bearing default parameters. This can be time-consuming depending on the size of the docking box and the computational power employed. To save time, if this calculation was already performed previously, the “grid” flag can be passed to BLinDPyPr so that the script does not needlessly run this step again. The grid.bmp and grid.nrg files must be placed on the working directory.

#### 2.2.4. Docking Run

**Figure 2:**
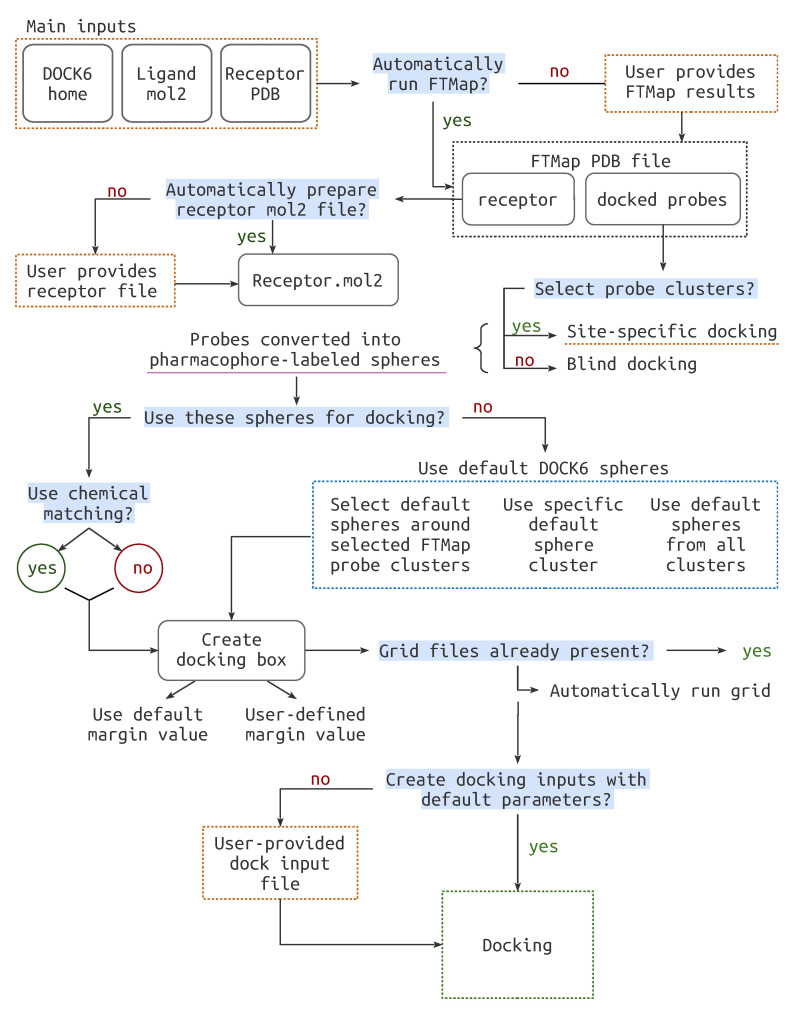
The complete workflow. for the BLinDPyPr program. Highlighted in blue are the decisions which must be made by the user. The files that may be provided to BLinDPyPr are marked with dashed orange lines.

By default, BLinDPyPr creates a docking input file containing the default parameters defined in the DOCK6 manual. However, the user can provide a custom input file. In this case, BLinDPyPr will only alter the parameters defined for the BLinDPyPr run. For instance, if chemical matching is to be used, BLinDPyPr will toggle this parameter in the custom input file, so that the user does not have to do so. BLinDPyPr writes a log file detailing all the steps performed in the docking run as well as all the parameters used to define it. The complete pipeline workflow can be visualized in Figure 2.

### 2.3. Benchmarking

PDBbind core version 2013 and Astex Diverse benchmarking sets were used to assess the pipeline’s pose prediction accuracy and compare it with the classic DOCK6 methods, also automated by BLinDPyPr. The receptor PDB files were manually edited to remove the redundant identical chains from the original crystal structures. They were then submitted to FTMap with default configuration parameters. The ligands were prepared with gasteiger charges and minimized using UCSF Chimera with 1000 steepest descent steps and 100 conjugate gradient steps. All benchmarking calculations were run serially on an Intel Core i9-9900K processor.

#### 2.3.1. PDBbind Core Set

To assess the performance of the novel BLinDPyPr pipeline methods with FTMap-derived spheres and compare those with classic docking methods, different protocols were explored with the PDBbind set:

- **FT+Chem:** Docking is performed on spheres derived from FTMap probe crossclusters, with chemical matching to further orient the ligands. This method was employed for blind and site-specific docking.
- **FT:** Docking is performed on spheres derived from FTMap probe crossclusters; however, chemical matching is turned off and the spheres pharmacophore labels do not influence pose prediction. This method was employed for blind and site-specific docking.
- **Manual site-specific:** It is a classic DOCK6 site-specific docking run. The accessory tool *sphgen* is used to generate classic spheres from a receptor surface file. DOCK6 sphere selector is then employed to select those in a radius of three Angstroms around the selected probe crossclusters,
- **Cavity-detection guided (Cluster 1):** It is a classic DOCK6 run using the first sphere cluster, defined by *sphgen* as the most likely to overlap with the real binding site.
- **Blind:** It is a classic DOCK6 blind run using all the generated spheres (sphere Cluster 0). This is the only case where DOCK6 must evaluate docking poses throughout the whole protein surface.

For each docking run, BLinDPyPr was prepared and run automatically with the default parameters described in Section 2.2, with two exceptions. In the test sets used to assess site-specific docking accuracy with the spheres derived from FTMap, we manually selected probe crossclusters present in the binding site. Moreover, for all the docking situations except Blind (Cluster 0), we altered the docking box default value to 10 Angstroms. This margin was increased in order to ensure that all ligands would fit in the docking box, thus avoiding possible growth errors for bigger ligands with many rotatable bonds.

Both FT and FT+Chem were separately benchmarked for site-specific and blind docking. To create the site-specific datasets, we visually selected only the FTMap probe clusters present in the protein binding site. The blind docking datasets were automatically prepared: by selecting no probe crossclusters, BLinDPyPr converts all docked probes into spheres. Table 1 describes the benchmarking groups and their variations.

The groups’ performances were assessed by calculating the RMSD, in Angstroms, between the docked poses of the ligands and their respective experimental conformations. Statistical analyses between the groups’ RMSD distributions were performed separately for the blind and site-specific categories using the Kruskal–Wallis method in conjunction with the Dunn post-hoc test. These calculations were performed using the JASP statistical software [28]. Furthermore, any RMSD under two Angstroms was considered a pose prediction success.

#### 2.3.2. Astex Diverse Set

This set was employed in order to further analyze the blind docking accuracy of BLinDPyPr. In the Astex studies, the protocols benchmarked were:

- **FT+Chem Blind**
- **FT Blind**
- **Cavity-detection Blind (Cluster 1)**

Therefore, the Astex set was employed to determine the accuracy of the new method of FTMap-derived spheres on the blind docking capacity, and to compare these with the results from classic DOCK6 cavity detection. Table 2 describes the benchmarking protocol groups for Astex in comparison to those employed with PDBbind.

## 3. Results and Discussion

### 3.1. PDBbind Core Set

**Figure 3:**
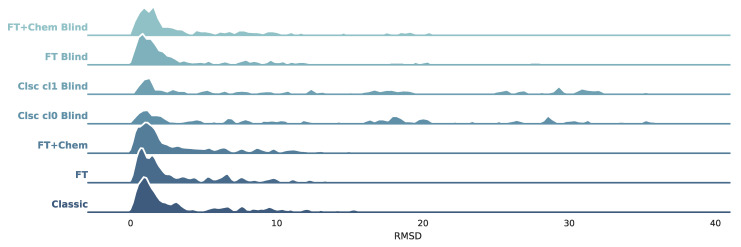
Kernel Density Estimate (KDE) plots for each benchmarking protocol.

All seven protocols described in Section 2.3.1 were tested against PDBbind’s protein–ligand complexes. As per the BLinDPyPr protocol, all receptors were submitted to FTMap. In eight of those, no FTMap probes could be found in the crystal ligand’s binding site, which made it impossible for those to be included in the site-specific benchmarking protocols. Furthermore, the classic DOCK6 benchmarking protocols require surface files for classic sphere generation, as explained in Section 2.2.3. As mentioned, this method tends to yield more errors since surface calculations may fail if the protein is very big and/or challenging. Therefore, classic protocols yielded fewer successful dockings than the protocols with FTspheres. For this reason, to ensure comparability, the final analyses were performed using only the protein–ligand complexes with successful dockings in all benchmarking protocols, totalling 157 complexes. A description of the RMSD distribution in all protocols can be found in Table 3 and it is visualized in Figure 3.

**Figure 4:**
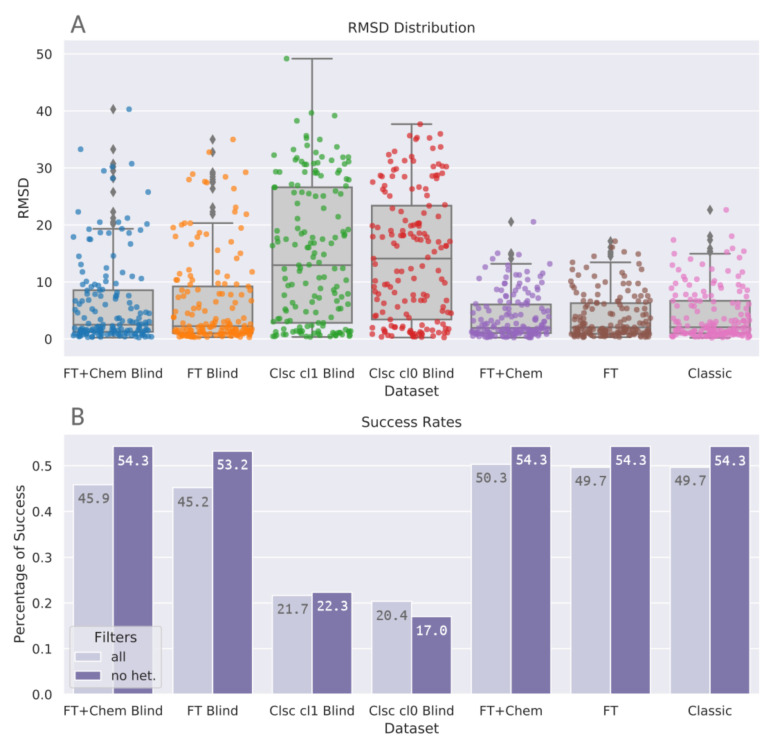
Summary of the benchmarking results with the PDBbind core set. (**A**) Boxplots combined with stripplots illustrate the distribution of the RMSD results for each benchmarking protocol. (**B**) Success rates at a 2.0 Å cutoff. Numbers printed inside the bars indicate success rates expressed in percentage rounded to three significant digits. Light bars (all) represent success rates for the whole datasets, while dark bars (no het.) represent success rates for the datasets with a filter to exclude receptors with nonstandard atoms.

Figure 3 shows that blind docking with classic DOCK6 methods with classic spheres (Clsc Cl0 Blind and Clsc cl1 Blind) achieve distributions which are more spread out if compared to site-specific protocols. This effect is less expressive on FT+Chem Blind and FT Blind. These novel methods with FTMap-derived spheres yield higher densities on lower RMSD values, achieving distributions similar to the non-blind datasets. Table 3 indicates the same trend: median RMSD values for FT+Chem Blind and FT Blind are 2.49 and 2.24, respectively, substantially closer to non-blind values (1.93, 2.04, and 2.06) than blind docking with classic spheres, whose median RMSD values were 12.9 for sphere Cluster 1 and 14.10 for sphere Cluster 0. Figure 4A condenses the distribution information, shown in Table 3 and Figure 3, and presents stripplots and boxplots for each protocol.

Success rates for top ranked poses were calculated, as shown in Figure 4B. In these analyses, two subsets of each benchmarking protocol were compared: (all) the original results, comprising the 157 previously mentioned protein–ligand complexes; and (no het.) a filtered subset which excludes receptors with nonstandard atoms in their structures. FTMap removes nonstandard atoms before docking the probes on the receptor surface. In this benchmarking study, BLinDPyPr uses this edited receptor file for the subsequent DOCK6 steps to ensure consistency between FTMap and docking results. Proteins with nonstandard atoms, especially those with such atoms located at the ligand binding site, will go through docking without them. However, the RMSD calculations are performed between the docked ligand (which was placed without regard to nonstandard atoms) and the experimental ligand, whose pose may have been influenced by the atom’s presence. To account for the negative impact this may exert on the success rates, we also calculate these rates for the filtered subset (no het.) which excludes proteins with nonstandard atoms. Another general concern is regarding the inverse correlation between docking accuracy and ligand size. To evaluate this, we separated the success rates into different categories of ligand size. In all ligand size categories, BLinDPyPr consistently had superior performance than classic blind approaches for all size categories analyzed (Figure S1).

There is no significant difference between protocols belonging to the site-specific docking category: FTMap derived spheres with chemical matching, without chemical matching, and classic DOCK6 spheres (*p* = 0.947, Kruskal-Wallis test). There is also no significant difference between FTspheres with chemical matching and its counterpart without chemical matching within the blind docking category (Table 4). However, there is a significant difference (*p* < 0.001) between blind docking with FTspheres and classic blind docking (Clusters 0 and 1) groups (Table 4). The Kruskal-Wallis test for the blind docking group returns *p* < 0.001 (statistic = 82.989). Figure 4 shows that docking using FTMap-derived spheres was able to elevate DOCK6 blind docking accuracy to the same level achieved by classic site-specific DOCK6 runs, therefore, the same accuracy yielded by site-specific classic DOCK6 runs can be expected from a blind docking run with the novel BLinDPyPr pipeline. Furthermore, the pipeline allows for evaluation of the best binding site for each candidate ligand, which makes it advantageous in a virtual screening campaign in which the receptor binding site is not exclusive.

To the best of our knowledge, the highest accuracy in the cavity detection-guided approach was achieved by Liu et al. with CB-dock [7], which yielded a success rate of 69.4% against Astex diverse set. Building from the conventional blind docking approach, Hassan et al. achieved with QuickVina-W [3] a 46% success rate of top ranked poses against PDBbind Core Set 2015. BLinDPyPr success rates for FT+Chem Blind and FT Blind through FTspheres in the no het. category were, respectively, 54.3% and 53.2%. This fits with BLinDPyPr’s initially intended purpose: in being a hybrid between the blind docking approaches, it also yields results intermediary to both.

### 3.2. Astex Diverse Set

BLinDPyPr was further benchmarked with the Astex Diverse set. In these analyses, only blind docking accuracy was assessed. For this reason, no verification was applied to the FTMap probes’ position in relation to the receptors’ binding sites. However, as described in Section 3.1, the final analyses were performed using only the protein–ligand complexes with successful dockings in all benchmarking protocols, totalling 75 out of the 85 protein–ligand complexes.

**Figure 5:**
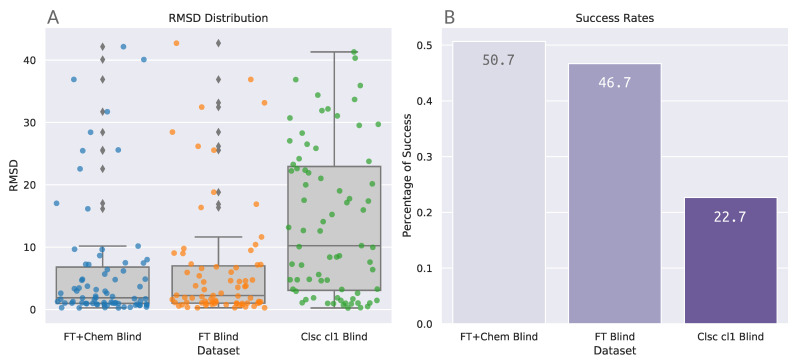
Summary of the benchmarking results with the Astex Diverse set. (**A**) Boxplots combined with stripplots illustrate the distribution of the RMSD results for each benchmarking protocol. (**B**) Success rates at a 2.0 Å cutoff. Numbers printed inside the bars indicate success rates expressed in percentage rounded to three significant digits.

The same trend previously identified with the PDBbind Set can also be observed in the Astex results (Table 5 and Figure 5): blind docking with FTspheres greatly reduces the median value in comparison to blind docking with DOCK6 sphere Cluster 1. Differently from what was observed for PDBbind, the FT+Chem Blind protocol was able to achieve a median value (1.87) lower than the established cutoff of 2.0 Å (Table 5). The Kruskal-Wallis test for these distributions returns *p* < 0.001 with statistic = 27.441. Post-hoc analyses for the Astex benchmarking group can be found in Table 6.

Figure 5A shows that indeed FTspheres protocols are able to achieve higher convergence of data points in lower RMSD values than the classic blind protocol. Success rates for unfiltered datasets FT+Chem Blind and FT Blind are 50.7% and 46.7%, respectively, also higher than the values achieved by the unfiltered datasets in the PDBbind analysis. The extension of benchmarking with the Astex Diverse Set reproduced and confirmed the blind docking accuracy trends determined initially through the PDBbind experiments.

### 3.3. Docking Elapsed Time

**Figure 6:**
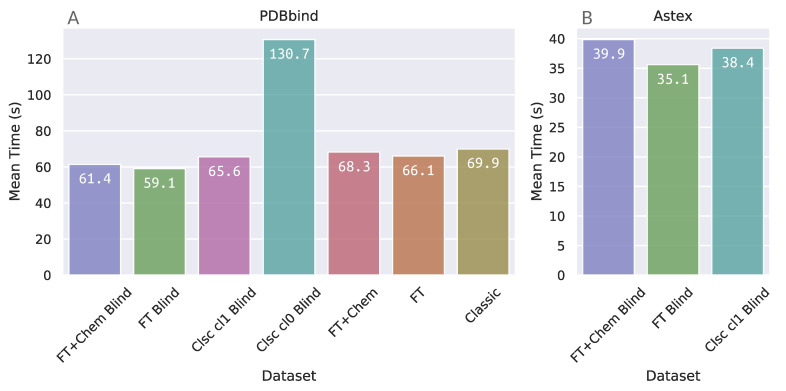
Mean elapsed time for each individual benchmarking protocol for docking in: (**A**) PDBbind Core Set; and (**B**) Astex Diverse Set.

The elapsed time for docking, as output by DOCK6, was extracted from every docking run in each benchmarking protocol. The mean elapsed time for docking on these datasets from PDBbind and Astex can be found in Figure 6. Dataset “Clsc cl0 Blind”, which corresponds to blind docking with DOCK6, demanded approximately double the running time as the other protocol datasets within the PDBbind group. This was expected, since the DOCK6 algorithm in this dataset is required to search the whole receptor surface.

Surprisingly, the FTsphere blind docking protocols demanded a mean amount of time that was very similar to the other datasets which are required to search in a more restricted docking box, even though FTsphere blind protocols compel DOCK6 to search for binding conformations in more than one potential binding site simultaneously.

On the other hand, although elapsed time for docking on FTsphere groups is similar to site-specific, the previous steps on the FTMap server and grid calculations (which are more computationally expensive for larger docking boxes) make the receptor preparation phase in these groups more time consuming in comparison to site-specific and classic sphere groups.

## 4. Conclusions

In this work, we present BLinDPyPr (Blind Ligand Docking through Preliminary Probing), a pipeline which associates automation and conversion scripts with well established programs such as FTMap and DOCK6 in order to introduce a hybrid approach to blind docking.

This method involves using the robust FTMap probing capabilities to guide DOCK6 to perform molecular docking on the specific regions defined by FTMap, restricting the search space to potential binding sites while allowing such sites to be simultaneously assessed for each candidate ligand.

The novel blind docking protocol’s accuracy was found to be similar to site-specific docking results achieved by classic DOCK6 runs, while expending the same mean time for docking. With FTMap guidance, our pipeline achieves 45.9% success rates on the PDBbind benchmarking set, while a classic DOCK6 blind docking run yields 20.4% and classic DOCK6 site-specific docking achieves 49.7%.

BLinDPyPr is an attractive alternative approach to virtual screening with larger ligand sets when such ligands need to be docked blindly or to receptors with more than one potential binding site, which need to be evaluated simultaneously.

## Figures and Tables

**Table 1:** Docking protocols employed in the PDBbind benchmarking studies (header) and the conditions with which they were tested (index row). CL, cluster; X, impossible combination.

	FT+Chem	FT	Classic	Classic CL0	Classic CL1
Site-specific	✓	✓	✓	✗	✗
Blind	✓	✓	✗	✓	✓

**Table 2:** Docking protocols employed in the Astex benchmarking studies (header) and the conditions with which they were tested (index row), in comparison with the protocol groups used with PDBbind. Black checkboxes: protocols performed with both Astex and PDBbind sets; Gray checkboxes, protocols performed with the PDBbind set only; CL, cluster; X, impossible combination.

	FT+Chem	FT	Classic	Classic CL0	Classic CL1
Site-specific	✓	✓	✓	✗	✗
Blind	✓	✓	✗	✓	✓

**Table 3:** Description of the distribution of RMSDs in each benchmarking protocol for PDBbind.

	FT+Chem Blind	FT Blind	Clsc cl1 Blind	Clsc cl0 Blind	FT+Chem	FT	Classic
count	157	157	157	157	157	157	157
mean	6.324	6.426	14.989	14.459	3.926	3.925	4.283
std	7.867	8.084	12.229	11.190	4.001	3.988	4.567
min	0.229	0.296	0.346	0.252	0.236	0.305	0.199
25%	1.252	0.997	2.822	3.411	1.042	0.916	0.997
50%	2.491	2.243	12.944	14.103	1.931	2.039	2.056
75%	8.549	9.211	26.607	23.375	6.050	6.276	6.692
max	40.300	34.995	49.174	37.683	20.547	17.142	22.646

**Table 4:** Dunn post-hoc test for the RMSD distributions between the benchmarking protocols belonging to the blind docking group in the PDBbind benchmarking studies.

Comparison	z	W${}_{i}$	W${}_{j}$	*p*	p${}_{bonf}$	p${}_{holm}$
Clsc cl0 Blind-Clsc cl1 Blind	0.069	381.140	379.736	0.473	1.000	0.848
Clsc cl0 Blind-FT Blind	6.570	381.140	246.599	<0.001	<0.001	<0.001
Clsc cl0 Blind-FT+Chem Blind	6.378	381.140	250.525	<0.001	<0.001	<0.001
Clsc cl1 Blind-FT Blind	6.502	379.736	246.599	<0.001	<0.001	<0.001
Clsc cl1 Blind-FT+Chem Blind	6.310	379.736	250.525	<0.001	<0.001	<0.001
FT Blind-FT+Chem Blind	−0.192	246.599	250.525	0.424	1.000	0.848

**Table 5:** Description of the distribution of RMSDs in each benchmarking protocol for Astex Diverse.

	FT+Chem Blind	FT Blind	Clsc cl1 Blind
count	75	75	75
mean	6.241	6.438	14.085
std	9.720	9.375	11.999
min	0.246	0.272	0.236
25%	0.958	1.005	3.065
50%	1.877	2.238	10.235
75%	6.804	6.996	22.936
max	42.148	42.713	41.307

**Table 6:** Dunn post-hoc test for the RMSD distributions between the benchmarking protocols on the Astex benchmarking studies.

Comparison	z	W${}_{i}$	W${}_{j}$	*p*	p${}_{bonf}$	p${}_{holm}$
Clsc cl1 Blind-FT Blind	4.277	145.013	99.553	<0.001	<0.001	<0.001
Clsc cl1 Blind-FT+Chem Blind	4.758	145.013	94.433	<0.001	<0.001	<0.001
FT Blind-FT+Chem Blind	0.482	99.553	94.433	0.315	0.945	0.315

## Supplementary Materials

The supplementary materials are available online.
