# Supplementary material for: Improving Blind Docking in DOCK6 through an Automated Preliminary Fragment Probing Strategy
Source: Molecules. 2021 Feb 25;26(5):1224. doi: 10.3390/molecules26051224 (PMC7956365; doi:10.3390/molecules26051224)
Supplement: Supplementary file 1 [file molecules-26-01224-s001.pdf]

Improving Blind Docking in DOCK6 through an Automated Preliminary Fragment Probing Strategy

Supplementary Material

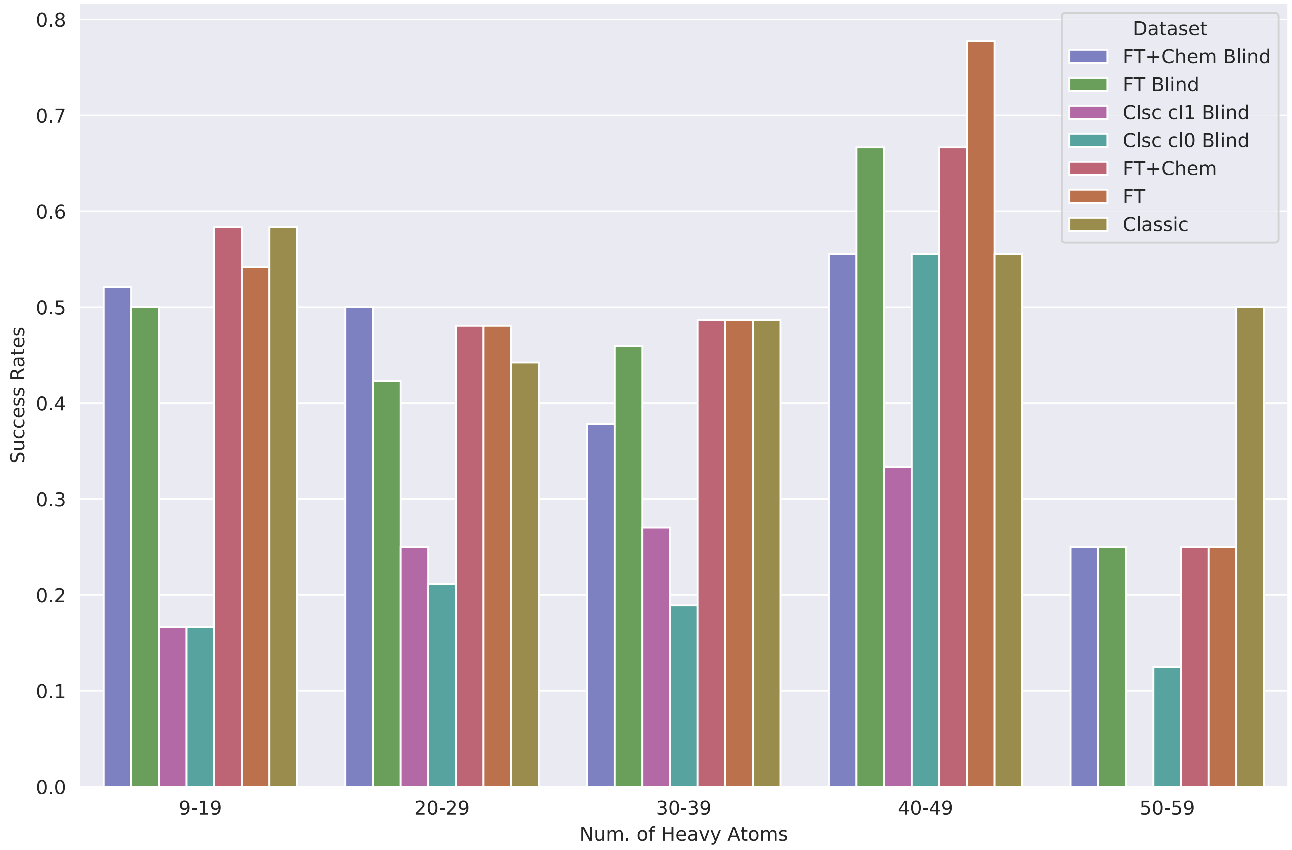

Figure S1: Distribution of success rates (RMSD < 2.0Å) for each PDBbind benchmarking protocol and for six different ligand size categories, distinguished by ligand number of heavy atoms. Minimum number of ligand heavy atoms was 9 and maximum was 70. Although we had 3 ligands in category 60-70, none were docked with RMSD < 2Å. The number of complexes in each category are: *9-19*: 48; *20-29*: 52; *30-39*: 37; *40-49*: 9; *50-59*: 8; *60-70*: 3.
